# Flexible High-Color-Purity Structural Color Filters Based on a Higher-Order Optical Resonance Suppression

**DOI:** 10.1038/s41598-019-51165-1

**Published:** 2019-10-17

**Authors:** Kyu-Tae Lee, Sung Yong Han, Zijia Li, Hyoung Won Baac, Hui Joon Park

**Affiliations:** 1 Department of Physics, Inha University, Incheon, 22212 South Korea; 2 Department of Electrical and Computer Engineering, Sungkyunkwan University, Suwon, 16419 South Korea; 3 Department of Energy Systems Research, Ajou University, Suwon, 16499 South Korea; 4 Department of Energy Science, Sungkyunkwan University, Suwon, 16419 South Korea; 5 Department of Organic and Nano Engineering, Hanyang University, Seoul, 04763 South Korea

**Keywords:** Optical materials and structures, Optical physics

## Abstract

We present flexible transmissive structural color filters with high-color-purity based on a higher-order resonance suppression by inserting an ultrathin absorbing layer in the middle of a cavity. A 3rd order Fabry–Pérot (F-P) resonance, which exhibits a narrower bandwidth than a fundamental F-P resonance, is used to produce transmissive colors with an improved color purity. The thin absorbing layer is properly placed at a center of the cavity to highly suppress only a 5th order F-P resonance appearing at a short wavelength range while not affecting the 3rd order F-P resonance for color generation, thus being able to attain the high-color-purity transmissive colors without reducing a transmission efficiency. In addition, angle-insensitive properties are achieved by compensating a net phase shift with a dielectric overlay and using a material with a high refractive index for the cavity medium. Moreover, the transmissive colors on a flexible substrate are demonstrated, presenting that changes in both the resonance wavelength and the transmission efficiency are nearly negligible when the color filters are bent with a bending radius of 5 mm and over 3000 times bending tests. The described approach could pave the way for various applications, such as colored displays, decorative solar panels, and image sensors.

## Introduction

Color filters have played a central role as a key element for a variety of applications, such as displays, image sensors, decorative solar panels, and light-emitting diodes (LEDs)^1–7^. Conventional color filters utilize colorant pigments, which rely on the light absorption to generate a desired color. Such light-absorbing characteristics of the conventional color filters cause a low efficiency of the resulting colors. Additional challenges of the existing color filters arise from their vulnerability to heat exposure, processing chemicals, moisture, and continuous ultraviolet (UV) illumination, causing a dramatic performance degradation over time^8^.

Structural color filters, which selectively transmit or reflect a certain proportion of visible light by a physical interaction between light and nanostructures, have attracted substantial attention for their potential in achieving improved efficiency, easy scalability, high resolution, and high stability. Various schemes of the structural color filters have been demonstrated by employing photonic resonances in thin-film multilayer structures and photonic crystals, perfect resonant absorptions in metamaterials and metasurfaces, and plasmonic resonances in nanocavities and metallic nanostructures at deep subwavelength scale^9–26^. However, most of the grating-based structural color filters present incident-angle-sensitive performances due to the momentum matching condition, which need to be addressed to be applied in practical applications. Non-trivial reflection phase shifts between semiconductors and metals have been exploited in both metal-dielectric-metal (MDM) thin-film and nanograting structure configurations, where a thickness of an optical cavity could be significantly decreased compared with the wavelength of incident light, so that a trivial change in the propagation phase shift was compensated by the reflection phase shifts^27–34^. Although the colors with the angle-invariant characteristics can be generated, the approach described above relies on the light absorption in a semiconductor layer with a high absorption coefficient, and therefore both the efficiency and the saturation remain poor. It has also been demonstrated that the angle-insensitive characteristics could be achieved by integrating a phase compensating dielectric overlay with a conventional Fabry–Pérot (F-P) cavity, where the net phase shift accumulated during a single round-trip remained nearly constant in wavelength over a broad range of incident angles^12,13^. This is because of the fact that the phase change accumulated during the propagation through the cavity medium is counteracted by the phase shifts occurring upon the reflection at the top and the bottom interfaces. However, a relatively thin metallic mirror needs to be employed to attain a high transmission efficiency over 60%, which causes the saturation to be highly affected due to a large amount of off-resonant wavelength components. The saturation can get improved by increasing the thickness of the metals in the cavity, which then notably reduces the efficiency of the transmitted colors. To resolve the above mentioned two challenges simultaneously, multicavity-resonance-based transmissive structural color filters featuring angle-insensitivity, high efficiency, and high saturation have been demonstrated^35^. The resonant wavelengths of the two different optical cavities were designed at a slightly shifted wavelength with optimized anti-reflection (AR) coatings on both sides so that the electric field in each cavity overlapped to remarkably boost the transmission efficiency without sacrificing the saturation, presenting over 60% transmission efficiency together with a greatly improved saturation for all individual colors. Despite a simple deposition process for the fabrication of the structural color filters, it requires a number of layers with a low thickness tolerance, because the thicknesses of the dual cavities need to be very carefully designed for the resonance overlap. Therefore, there is a critical necessity to develop a new color filtering strategy that can overcome the aforementioned challenges.

In this work, we demonstrate multilayer semitransparent structures for producing transmissive colors with high-color-purity, angle-insensitivity, and high efficiency, based on a higher-order resonance suppression. Although employing a 3rd order F-P resonance leads to a narrowband transmission in a spectral curve of transmittance as compared to a fundamental F-P resonance for achieving the high-color-purity transmissive colors, a 5th order F-P resonance appears at a short wavelength regime when the 3rd order F-P resonance is utilized for color generation, which significantly degrade the color purity. To address this challenge, the structural color filters are designed to have an electric field intensity profile with a maximum value of the 5th order resonance but a minimum value of the 3rd order resonance at the center of the cavity so that only the 5th order resonance is significantly suppressed without affecting the 3rd order resonance by introducing a lossy medium in a middle of the cavity. This guarantees the high-color-purity transmission color generation without sacrificing the transmission efficiency. Besides, a resonant wavelength of the individual structural color filters remains almost constant in wavelength over a broad range of incident angles up to 60°, due to a phase compensation by a dielectric overlay and a small refraction angle by a cavity material with a high index of refraction. Additionally, the structural color filters, fabricated on a flexible substrate, present insensitive performances against the number of a bending deformation and a bending radius. The present concept can be easily enabled by a simple deposition method, which can offer a key step toward the realization of the large-scale applications in a variety of research fields, including as e-paper display technologies, image sensors, flexible optoelectronic devices, and decorations.

## Results and Discussion

**Figure 1:**
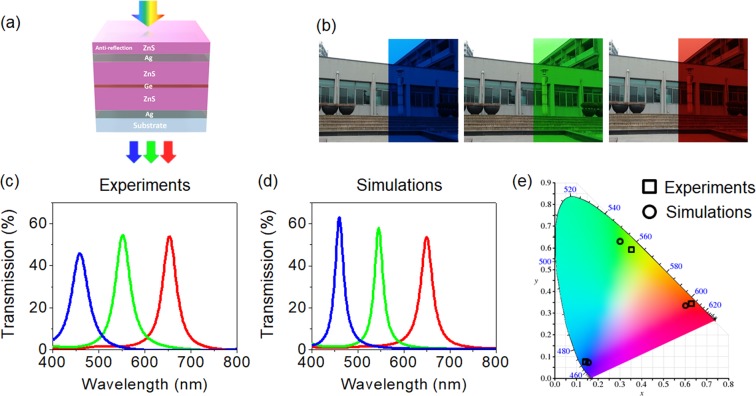
(**a**) Schematic diagram of transmissive structural color filters with wide angle, high efficiency, and high saturation, based on a higher-order resonance suppression. (**b**) Optical images of fabricated transmissive structural color filters on a glass substrate. (**c**) Measured and (**d**) simulated transmission spectra of the structural color filters at normal incidence. (**e**) Color coordinates evaluated from the measured (squares) and the simulated (circles) transmission spectra, described on the CIE 1931 chromaticity diagram.

Figure 1(a) depicts a schematic diagram of multilayer structural color filters creating vivid transmissive colors with the improved color purity, angle-invariant performances, and high efficiency, by introducing an absorbing medium in the middle of the cavity to suppress the higher-order resonances. The structural color filters comprise a transparent dielectric material sandwiched by high reflecting mirror surfaces with a phase compensating dielectric overlay. The center cavity layer, which is designed to form the 3rd order F-P resonance, is divided into two layers with the same thickness, where an ultrathin lossy semiconductor film is embedded between two separate cavity media for selectively eliminating the 5th order F-P resonance at the shorter wavelength range. For the optical cavity medium, zinc sulfide (ZnS) was selected because its refractive index in the visible range is pretty high with a negligible absorption, thus allowing the angle of refraction to be trivial at non-normal incident angles for achieving the angle-insensitive property. The dielectric overlayer was added onto the structure to provide a phase compensation, contributing to the angle-insensitive performance, and ZnS was also utilized for simplicity^35^. Additionally, this overlayer works as the AR coating, suppressing the reflection at a certain wavelength for better transmittance. Silver (Ag) was chosen for the metal mirrors since it has the highest reflectivity with the lowest absorption in the visible regime, while germanium (Ge) was selected for the ultrathin lossy medium, which is placed in a proper position of the cavity to selectively suppress the higher-order resonance. The device configuration is ZnS/Ag/ZnS/Ge/ZnS/Ag/substrate. Note that other semiconductors, such as silicon (Si) and SiGe, and other lossy metals, such as nickel (Ni), titanium (Ti), tungsten (W), and chromium (Cr), can be used for the absorbing medium at the center of the optical cavity. We also note that other wide-bandgap semiconductors, such as titanium dioxide (TiO_2_), tantalum pentoxide (Ta_2_O_5_), zinc oxide (ZnO), tungsten trioxide (WO_3_), vanadium pentoxide (V_2_O_5_), and molybdenum trioxide (MoO_3_), can replace the transparent cavity medium with the high refractive index. Figure 1(b) shows photographs of the structural color filters fabricated on a glass substrate over a large area with a size of 4 × 4 cm, displaying that a background building can be obviously observed through the fabricated devices with the vivid red (R), green (G), and blue (B) colors. Such a large area fabrication is easily enabled due to the fact that only the deposition method is involved for the fabrication of the color filter devices, which will be demonstrated on a flexible substrate later. Figure 1(c) exhibits measured transmission spectra at normal incidence, which match well with simulated transmission spectra as displayed in Fig. 1(d). It is clear that the sharp resonances with greatly suppressed off-resonance wavelengths and with the transmission efficiency over 50% are obtained, both of which are very important for the color filters. A transfer matrix method-based optical simulation is conducted with refractive indices characterized by using a spectroscopic ellipsometer (Elli-SE, Ellipso Technology Co.), which are provided in Fig. S1. The measured spectral curves of transmittance of the structural color filters are attained by utilizing a spectrometer (V-770 UV-Visible-Near Infrared Spectrophotometer, JASCO). 110 (30), 90 (35) and 70 (35) nm of ZnS (Ag) are used for the cavity layer (mirror) for the R, G, and B transmissive color generation, respectively. Thicknesses of the optimized dielectric overlays are found to be 55, 45, and 35 nm for the R, G, and B colors, respectively. 5 nm of Ge is inserted between two 70 nm (92 nm)-thick ZnS cavities in the middle for the B color (G color), while 13 nm of Ge is placed between two 112 nm-thick ZnS cavities for the R color. The thicknesses of each layer for the RGB colors are summarized in Table 1. The multiple thin-films of ZnS, Ag, and Ge are deposited on the glass substrate using electron-beam evaporation. Atomic force microscopy (AFM) on the surface of both ZnS (45 nm) and Ag (35 nm) on the Si substrate, where ZnS and Ag were deposited by using the e-beam evaporator, was conducted (Fig. S2). It was found that the root mean square values, which are defined as the standard deviation of the surface height profile from the average height, are 0.743 nm and 1.425 nm for ZnS and Ag, respectively, both of which can be regarded as a smooth surface. X-ray diffraction (XRD) was also performed on the ZnS surface, presenting that a sharp peak (2θ ≈ 28.5°), which indicates that the film is a crystalline structure (Fig. S3). The measured spectral curves of transmittance exhibit the resonance with 54.0%, 54.6%, and 46.9% of the transmission efficiency at 653, 552, and 459 nm for the RGB colors, all of which are in good agreement with the simulated profiles that show the resonance with 53.7%, 58.1%, and 63.1% of the efficiency at 650, 545, and 460 nm for the RGB colors, respectively. Both the full width at half maximum (FWHM) and the transmission efficiency of the structural color filters obtained from both experiments and simulations are summarized in Table 2. In Fig. 1(e), color spaces (*x*, *y*) computed from both the measured and the simulated spectral curves of transmittance are illustrated on the CIE 1931 chromaticity diagram to estimate the purity of the transmissive colors of the structural color filters. The color spaces obtained from the measured transmission spectra represented by black squares are found to be (0.626, 0.341), (0.351, 0.594), and (0.139, 0.077), whereas (0.602, 0.333), (0.300, 0.626), and (0.150, 0.076) are attained from the simulated transmission profiles denoted by black circles for the RGB colors, respectively. The color triangle defined by the RGB color space obtained from the color filter devices is wide, which means that a wide range of the transmissive colors can be generated. It is important to note that the gamut of the RGB color space achieved by the structural color filters proposed in this paper is comparable to that of the standard RGB color space of the display, which are (0.640, 0.330), (0.300, 0.600), and (0.150, 0.060) for the RGB colors, respectively. It is worth noting that the transmission efficiency of the structural color filters can be further improved up to ~80% by adding an optimized AR coating layer at the bottom of the device. Both the thickness and the refractive index of the bottom AR layer are optimized and provided in Fig. S4. The transmission spectra and surface admittance analyses without and with the bottom AR layer are given in Figs. S5 and S6, respectively.

**Table 1:** Thicknesses of each layer for the RGB colors.

	Blue	Green	Red
Top ZnS	35 nm	45 nm	55 nm
Top Ag	35 nm	35 nm	30 nm
ZnS cavity	140 nm	185 nm	225 nm
Ge	5 nm	5 nm	13 nm
Bottom Ag	35 nm	35 nm	30 nm

**Table 2:** Full width at half maximum (FWHM) and transmittance of each color.

	FWHM (nm)		Transmittance (%)	
	Experiments	Simulations	Experiments	Simulations
Blue	44	24	46.9	63.1
Green	38	22	54.6	58.1
Red	36	31	54.0	53.7

**Figure 2:**
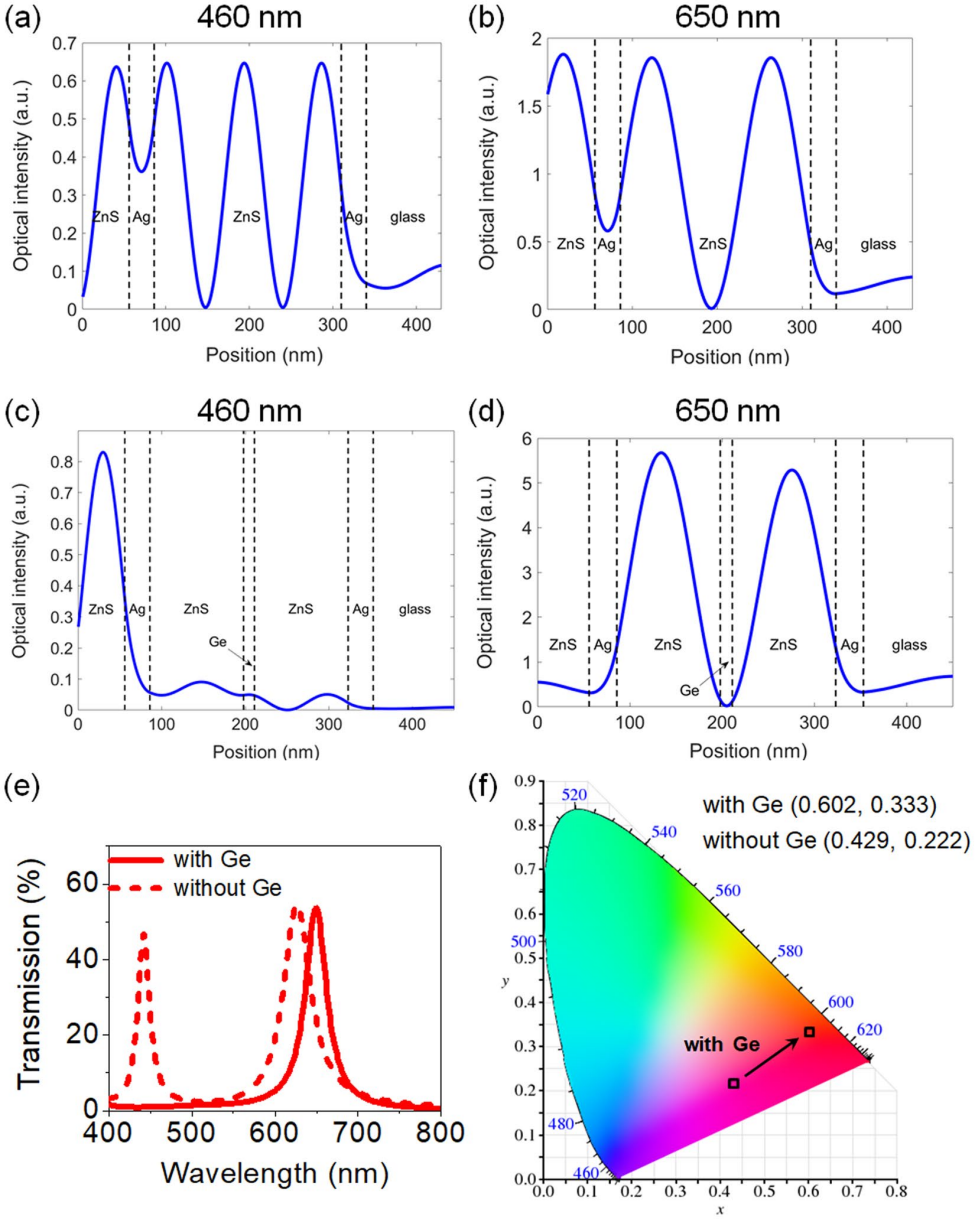
Intensity profiles of the electric field of the transmissive structural color filters without the ultrathin light-absorbing intermediate layer at (**a**) 460 nm and (**b**) 650 nm, and with the ultrathin light-absorbing intermediate layer at (**c**) 460 nm and (**d**) 650 nm. The absorbing layer inserted in the middle of the cavity greatly suppresses only the 5th order F-P resonance at 460 nm but doesn’t affect the 3rd order F-P resonance at 650 nm. (**e**) Simulated transmission spectra and (**f**) the corresponding chromaticity diagrams of the red structural color with (solid) and without (dashed) the light-absorbing intermediate Ge layer.

Figures 2(a,b) show the electric field intensity profiles in the R colored filter without the ultrathin absorbing medium in the middle of the cavity at 460 and 650 nm, respectively. As can be seen from the figures, the structural color filter without the intermediate lossy medium shows a strong electric field intensity in the middle of the cavity for the 5th order F-P resonance at 460 nm, but a nearly zero electric field intensity in the middle of the cavity for the 3rd order F-P resonance at 650 nm. It is thus expected that only the 5th order F-P resonance can be significantly suppressed by putting the ultrathin absorbing layer in the middle of the two ZnS cavities without affecting the 3rd order F-P resonance, indicating that the purity of the R color can be markedly improved by only absorbing the 5th order F-P resonance without reducing the transmission efficiency. In Figs. 2(c,d), the intensity distributions of the electric field into the structure with the ultrathin intermediate absorbing Ge layer at 460 and 650 nm are provided. Although the electric field at 650 nm is not influenced by the additional lossy medium, which is important to maintain the high transmission efficiency of the R color, the electric field at 460 nm is strongly mitigated, which is critical to accomplish the high color purity. This can be clearly seen in the simulated transmission spectra presenting that the 5th order F-P resonance appearing at 460 nm is greatly suppressed by inserting the ultrathin Ge layer without sacrificing the efficiency of the 3rd order F-P resonance at 650 nm, as shown in Fig. 2(e). Note that the effective refractive index of the cavity medium after introducing the intermediate Ge layer gets higher than that of the cavity without the Ge layer, thus slightly shifting the resonance toward the longer wavelength region. We also note that the wavelengths of the 1st, 3rd, and 5th F-P resonances are found to be ~270 nm, ~ 460 nm, and ~970 nm for the blue, ~330 nm, ~545 nm, and ~1120 nm for the green, and ~370 nm, ~650 nm, and ~1405 nm for the red, respectively. The corresponding chromaticity diagram is displayed in Fig. 2(f), where the color spaces for the R color filter without and with the ultrathin light-absorbing Ge layer are found to be (0.429, 0.222) and (0.602, 0.333), respectively, thus validating much improved color purity after inserting the Ge layer in the middle of the cavity. The effects of the thickness of Ge on the optical properties and the corresponding chromaticity of the structural color filters are presented in Figs. S7(a,c), and those depending on the kind of material for light-absorbing layer are depicted in Figs. S7(b,d). Additionally, optical properties, color purity, and the electric field distributions of the structural color filters based on the 3rd and 5th order resonances are compared to those of the structural color filters employing the 1st and 3rd order resonances, which are given in Figs. S8 and S9. As expected, it could be observed that the transmission efficiency of the 1st order resonance was greatly reduced with the ultrathin light-absorbing Ge layer, degrading its performance significantly.

**Figure 3:**
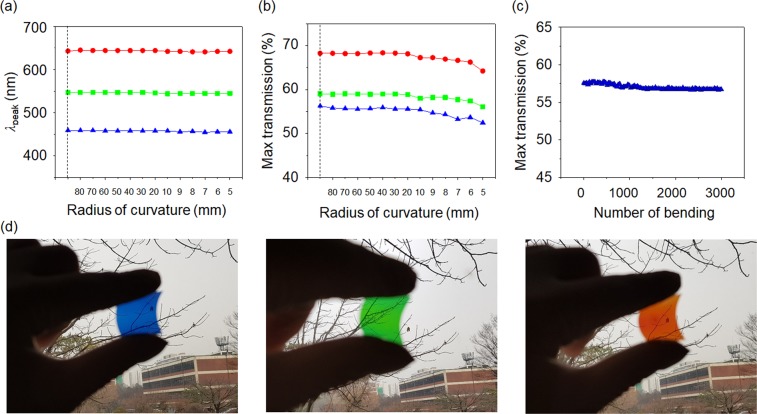
(**a**) Positions of a resonant wavelength and (**b**) maximum values of a transmission efficiency of the fabricated transmissive structural color filters on a plastic substrate as a function of a radius of curvature. (**c**) Maximum values of the transmission efficiency of the blue structural color against the number of bending deformation tests. (**d**) Optical images of the fabricated transmissive structural color filters on the flexible substrate.

Due to the simplicity of the device fabrication, which only involves the thin-film deposition method, the structural color filters demonstrated in this work can be easily fabricated on a flexible substrate. After the fabrication of the structural color filters on the polyethylene terephthalate (PET) substrate, their performance variations depending on the bending radius of curvature (ranging from 80 to 5 mm) and the number of the bending test (up to 3,000 times) are explored to evaluate the long-standing characteristics of the flexible structural color filter. Figures 3(a,b) depict the measured peak wavelength positions and the maximum transmission efficiencies of the colored devices as a function of the radius of curvature of the plastic substrate, both of which exhibit that both the resonant wavelengths and the peak efficiency values are maintained even with 5 mm of the bending radius. Figure 3(c) shows the maximum value of the transmission efficiency of the B colored device, measured by deforming the flexible color filter with multiple bending tests, and the structural color filter shows the insensitive performance with respect to the bending deformation up to 3,000 bending tests, thereby opening the possibility of electronic paper displays and ultrathin flexible/foldable/wearable displays. The measured and the simulated spectral curves of transmittance of the flexible RGB color filters are described in Fig. S10. Figure 3(d) provides photographs of the fabricated flexible structural RGB color filters, presenting that the background can be readily perceived through the devices with the distinct RGB transmissive colors.

**Figure 4:**
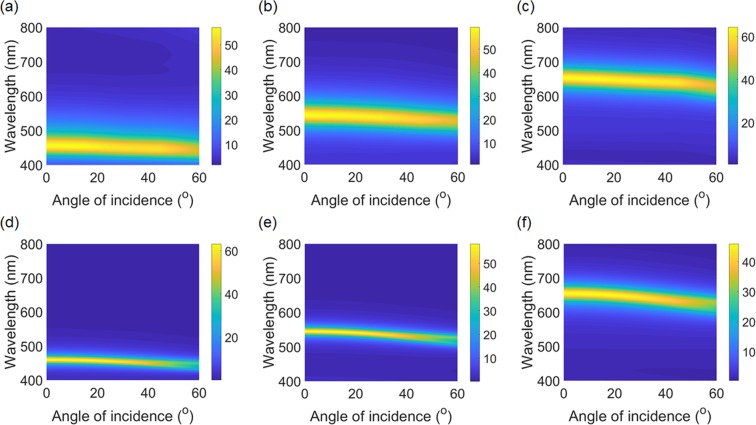
(**a**–**c**) Measured and (**d**–**f**) simulated angle-resolved transmission spectra of the transmissive structural color filters under the unpolarized light illumination, showing that the resonant wavelengths remain nearly constant in wavelength over a wide incident angle range up to 60°.

As a real part of the refractive index of ZnS is relatively high (2.342 at 545 nm) with a negligible imaginary part in the visible wavelength region, the refraction angle into the ZnS cavity at non-normal incidence is reduced (*e.g*., refraction angle is ~21.7° at 545 nm when the incidence angle is 60°). This means that a change in the net phase shift (*i.e*., round-trip propagation phase shift + two reflection phase shifts at both top and bottom interfaces) is mitigated and thus the resonance wavelength remains nearly constant. In addition to the reduced refraction angle, it has been demonstrated that a top dielectric overlay provides proper phase compensation to further improve the angular sensitivity. These two are the main reasons behind the angle-insensitive performance^12,13,35^. Figs. 4(a–c) present measured angle-resolved spectral curves of transmittance of the structural color filters, where a sharp resonance in the transmission spectrum is retained over a wide angle of incidence up to 60° for unpolarized light illumination. The simulated angle-resolved profiles shown in Figs. 4(d–f) agree well with the measured results. In addition to the small refraction angle in the high index cavity medium, it is important to note that a dielectric overlay with the half thickness atop the structure provides a phase compensation, which also contributes to the angle-insensitive performance, as mentioned earlier. The dielectric overlay also functions as the AR coating reducing the reflection at a certain wavelength.

## Conclusion

In summary, we have demonstrated flexible structural transmissive colors featuring wide angle, high efficiency, and high saturation, exploiting the 3rd order F-P resonance at resonant wavelengths with an efficient suppression of the 5th order F-P resonance at shorter wavelengths. The ultrathin light-absorbing layer is placed in the middle of the optical cavity, where the structural color filters show a maximum intensity of the electric field for the 5th order F-P resonance but a minimum intensity of the electric field for the 3rd order F-P resonance, thereby yielding a considerable suppression of only the 5th order F-P resonance and a significantly improved saturation without lowering the transmission efficiency. Moreover both high refractive index of the cavity medium and the phase compensating dielectric overlay allow the resonant wavelengths to remain almost constant for a wide angle of incidence up to 60°. Furthermore, the optical properties of the flexible structural transmissive colors are found to be pretty insensitive to both the bending radius and the number of the bending deformation. The strategy described in this work could offer the distinct potentials to achieve diverse large-scale applications, such as flexible display devices, wearable electronics, imaging devices, decorations, and colored solar cells.

## Methods

### Device fabrication

Device structures, composed of multiple thin-film layers of ZnS, Ag, and Ge (ZnS/Ag/ZnS/Ge/ZnS/Ag/substrate) were fabricated by consecutive E-beam evaporation processes on glass or PET substrates.

### Simulations and measurements

Simulations based on the transfer matrix method were carried out. The transfer matrix method is based on the continuity of the electric field across the interfaces between two homogeneous media having different index of refraction. The electric field in the successive medium can be derived through a matrix operation if the electric field at the first layer is known. Reflection and transmission coefficients can be calculated from the matrix for the entire system. The transmission spectra at normal incidence, angular performances, and the electric field intensity distributions into the color filter structures, where the measured refractive indices of Ag, ZnS, and Ge were used, are investigated by using the transfer matrix method based simulations. The refractive indices of these materials were measured using a spectroscopic ellipsometer (Elli-SE, Ellipso Technology Co.). Measured transmission spectra at normal and non-normal angles of incidence were obtained by using a spectrometer (V-770 UV-Visible-Near Infrared Spectrophotometer, JASCO).

## Supplementary information

Supplementary Information
