# Supplementary material for: Flexible High-Color-Purity Structural Color Filters Based on a Higher-Order Optical Resonance Suppression
Source: Sci Rep. 2019 Oct 17;9:14917. doi: 10.1038/s41598-019-51165-1 (PMC6797723; doi:10.1038/s41598-019-51165-1)
Supplement: Supplementary file 1 — Supplementary Information [file 41598_2019_51165_MOESM1_ESM.pdf]

Supplementary Information

Flexible High-Color-Purity Structural Color Filters Based on a Higher-Order Optical Resonance Suppression

Kyu-Tae Lee[1,2,†], Sung Yong Han[3,†], Zijia Li[4,5], Hyoung Won Baac[2,*] & Hui Joon Park[5,*]

[1]Department of Physics, Inha University, Incheon 22212, South Korea

[2]Department of Electrical and Computer Engineering, Sungkyunkwan University, Suwon 16419, South Korea

[3]Department of Energy Systems Research, Ajou University, Suwon 16499, South Korea

[4]Department of Energy Science, Sungkyunkwan University, Suwon 16419, South Korea

[5]Department of Organic and Nano Engineering, Hanyang University, Seoul 04763, South Korea

* Prof. Hui Joon Park (huijoon@hanyang.ac.kr)

Prof. Hyoung Won Baac (hwbaac@skku.edu)

[†] These authors contributed equally to this work.

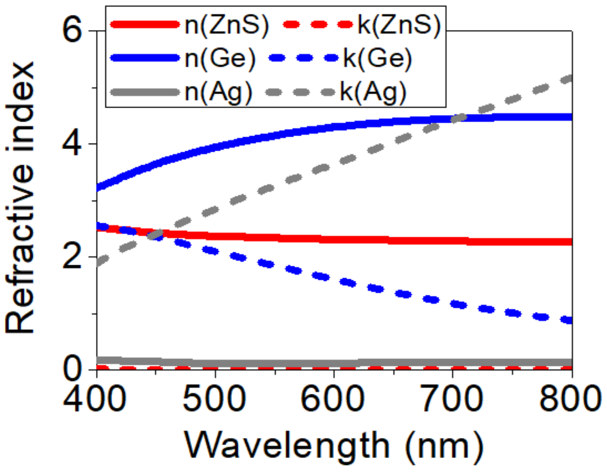

Figure S1. Refractive indices of ZnS, Ge, and Ag, measured by using a spectroscopic ellipsometer (Elli-SE, Ellipso Technology Co.).

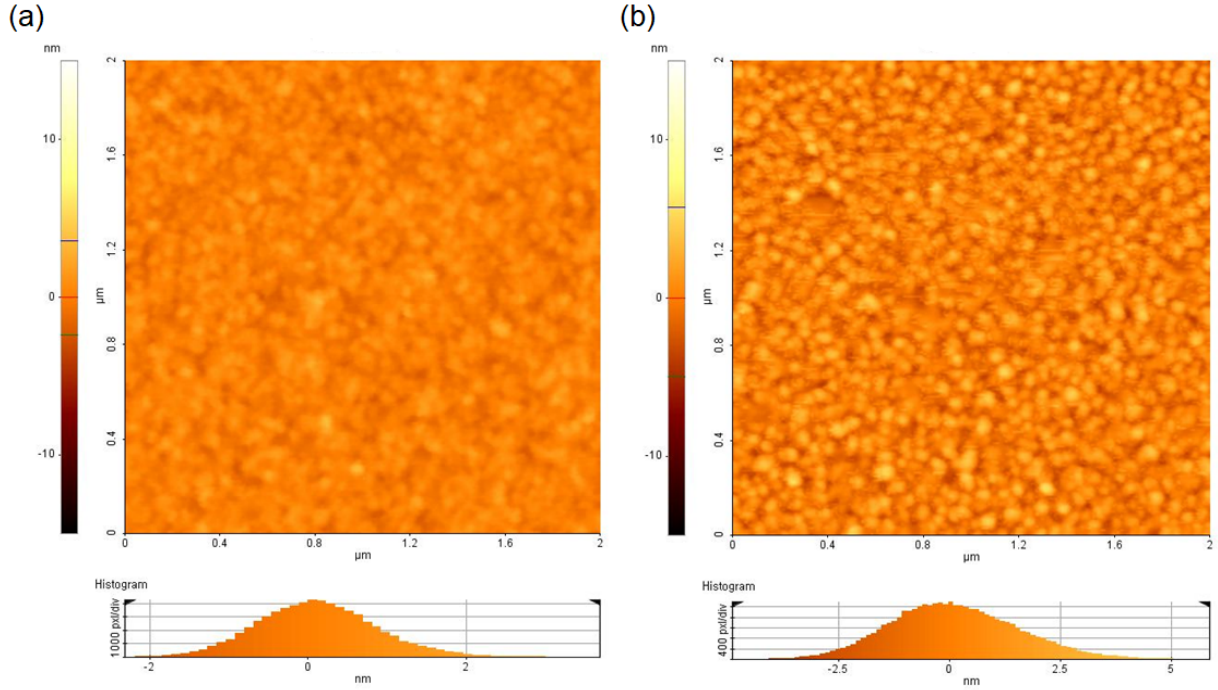

Figure S2. Atomic force microscopy (AFM) images of (a) 45 nm-thick ZnS and (b) 35 nm-thick Ag on the Si substrate (XE100, Park Systems, Korea).

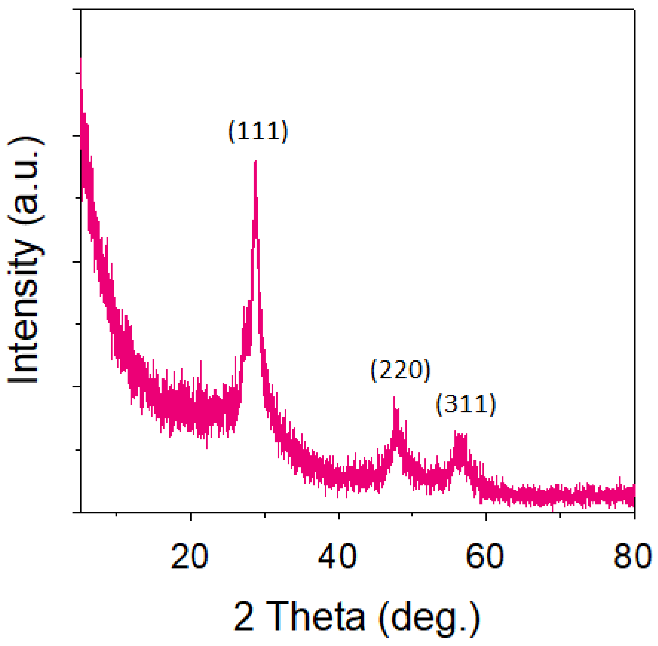

Figure S3. X-ray diffraction (XRD) pattern of ZnS (Ultima III, Rigaku, Japan).

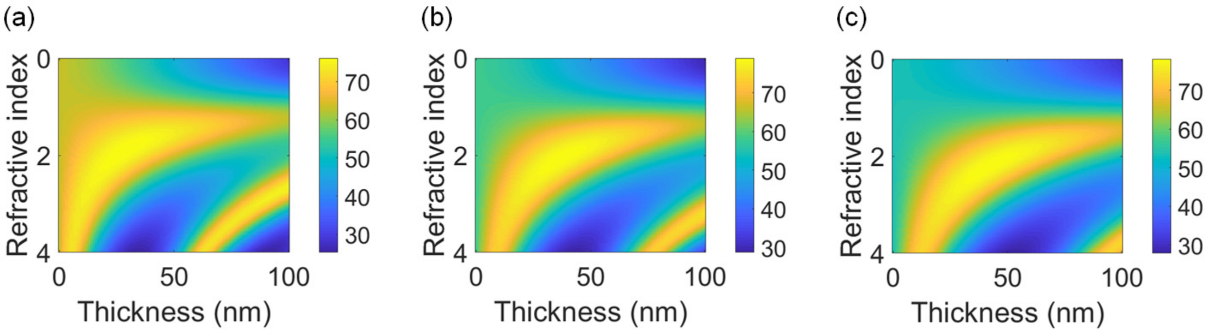

Figure S4. 2D contour plots of transmission as a function of a thickness and a refractive index of a bottom anti-reflection layer for (a) blue (B), (b) green (G), and (c) red (R) colors at 460, 545, and 650 nm, respectively.

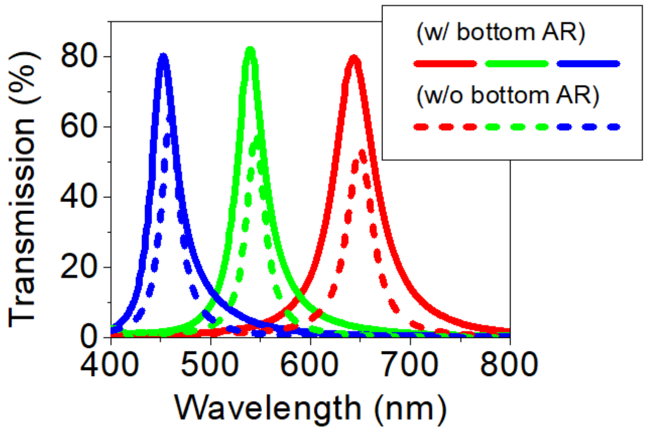

Figure S5. Simulated transmission spectra with (solid lines) and without (dashed lines) the bottom anti-reflection coating.

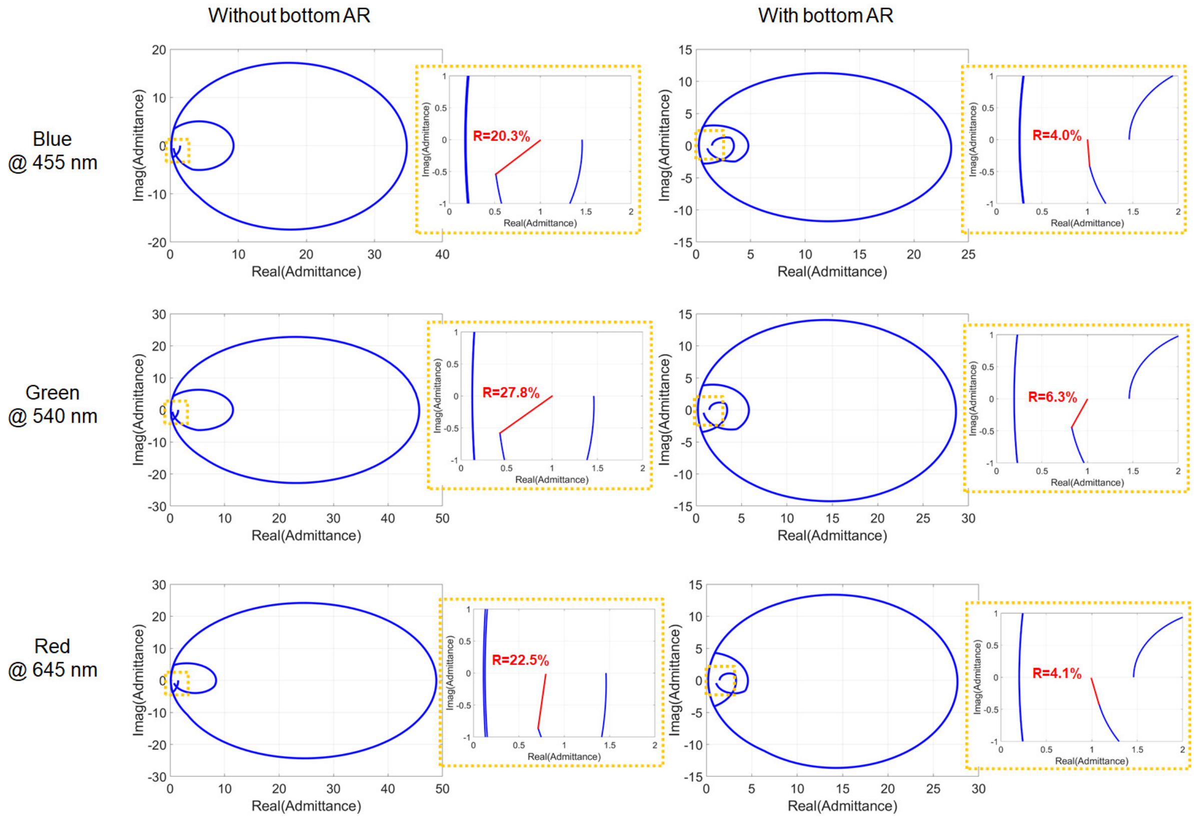

Figure S6. Admittance diagrams without (left) and with (right) the bottom anti-reflection coating for the RGB colors. Insets show an expanded view of each case along with a calculated reflectance at the resonant wavelengths.

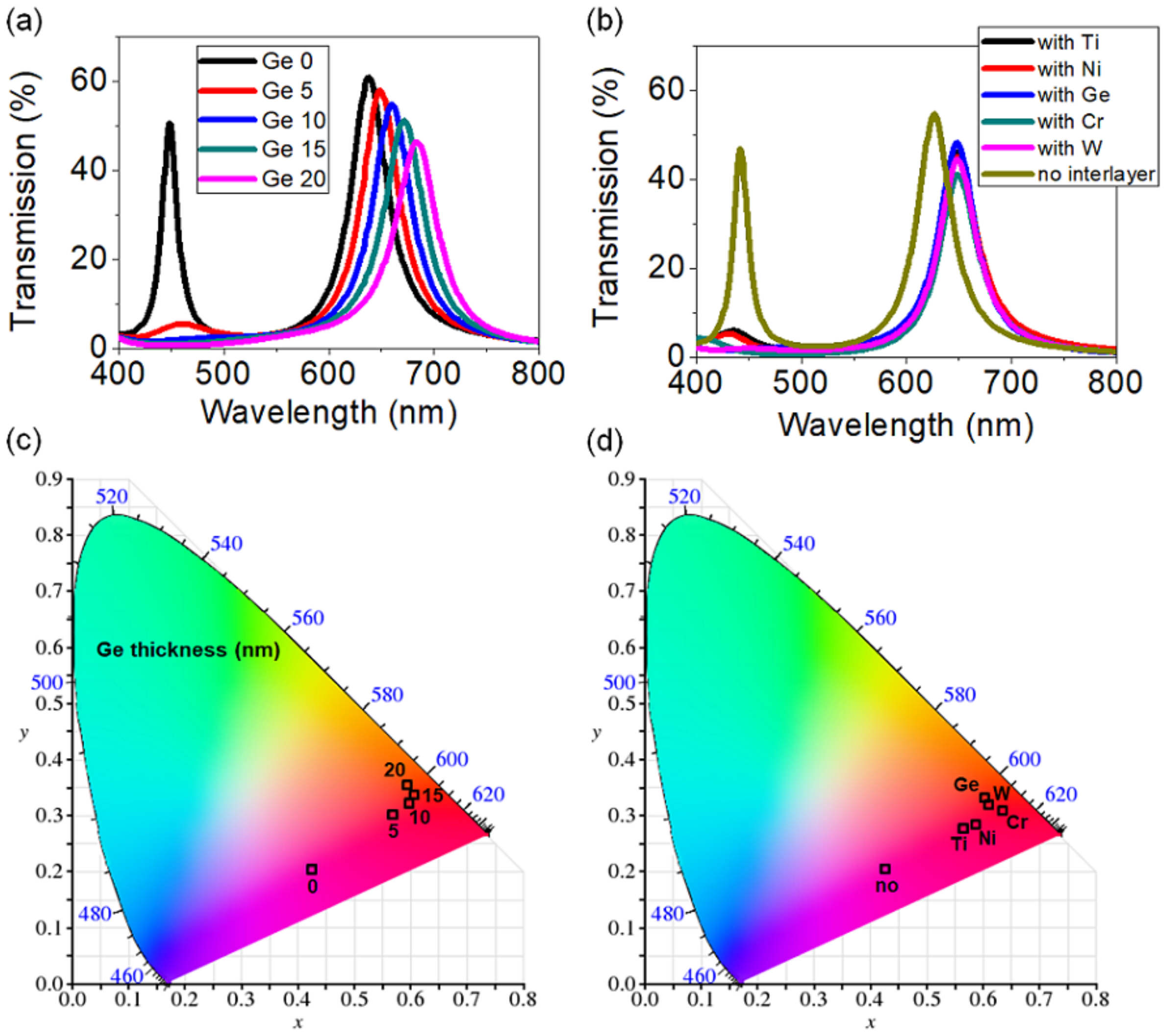

Figure S7. Simulated transmission spectra and the corresponding color purity depending on a thickness of Ge and different light-absorbing materials.

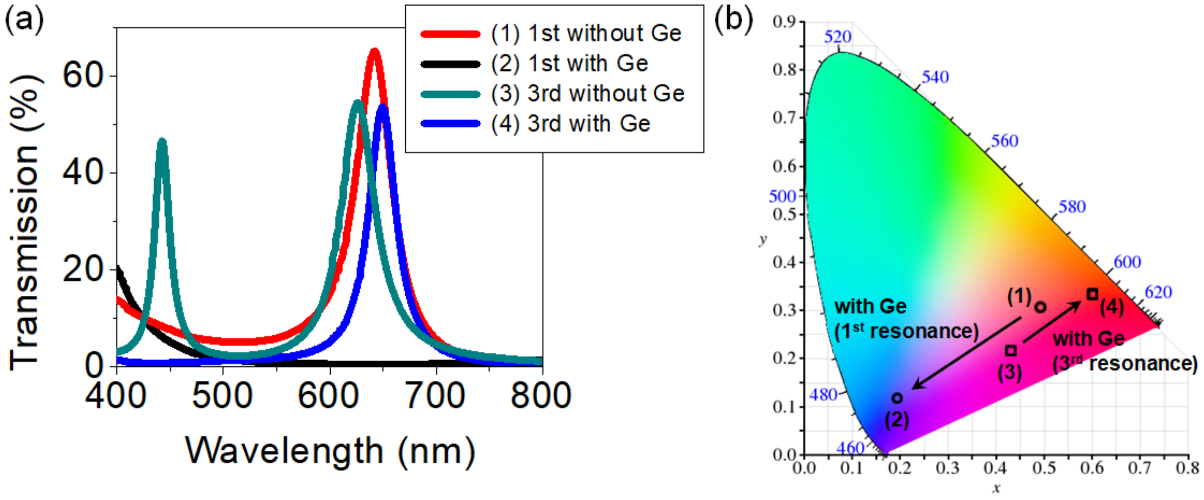

Figure S8. Simulated transmission spectra and the corresponding color purity of the structural color filters based on 1st and 3rd order resonances, and 3rd and 5th order resonances.

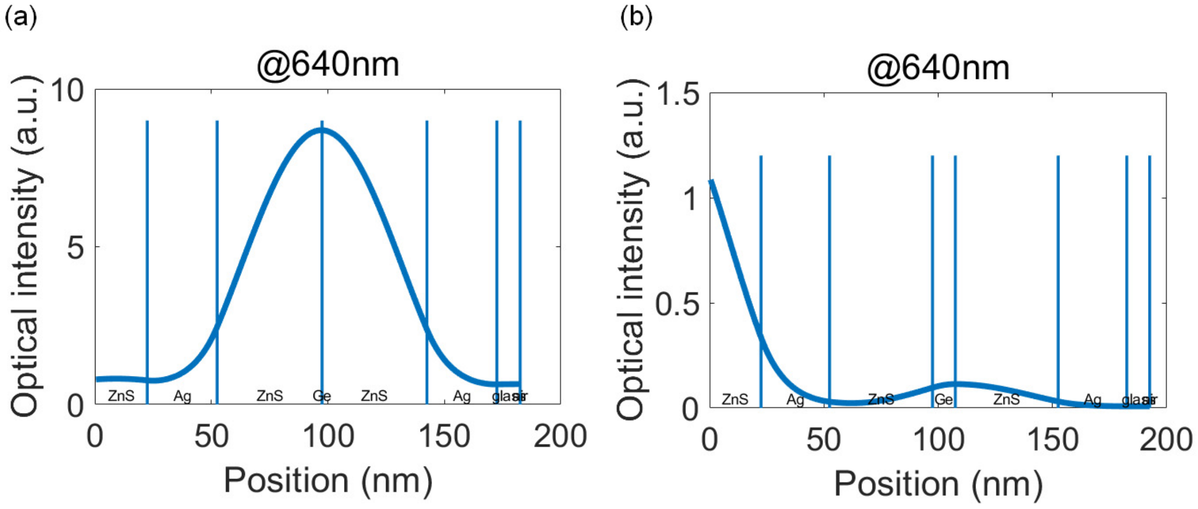

Figure S9. Electric field distributions at 640 nm for the red structural color filter employing 1st order resonances.

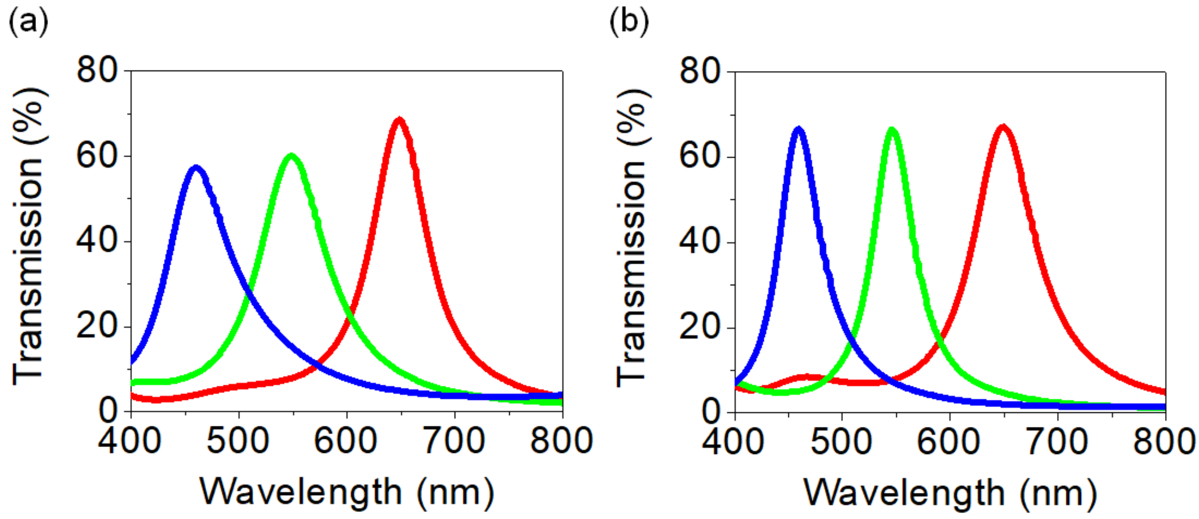

Figure S10. (a) Measured and (b) simulated transmission spectra of the flexible structural color filters at normal incidence.
